# History of Inflammatory Bowel Diseases

**DOI:** 10.3390/jcm8111970

**Published:** 2019-11-14

**Authors:** Giovanni Clemente Actis, Rinaldo Pellicano, Sharmila Fagoonee, Davide Giuseppe Ribaldone

**Affiliations:** 1 Gastroenterology and Clinical Immunology, Turin General Hospitals, 10100 Turin, Italy; actis_g@libero.it; 2 Unit of Gastroenterology, Molinette Hospital, 10123 Turin, Italy; 3 Institute of Biostructures and Bioimaging (CNR) c/o Molecular Biotechnology Center, 10126 Turin, Italy; sharmila.fagoonee@unito.it; 4 Department of Surgical Sciences, University of Turin, 10100 Turin, Italy; davrib_1998@yahoo.com

**Keywords:** ulcerative colitis, inflammatory bowel disease, therapy, inflammation, bowel, interactome

## Abstract

Inflammatory bowel diseases (IBD) are characterized by chronic inflammation of the intestinal mucosa and unknown etiology. In this review, we identified three main eras in the IBD history. Between the 19th and the 20th century, the primary task had been the definition of the diagnostic criteria in order to differentiate the new entity from intestinal tuberculosis. In the 20th century, an intense and prolific therapeutic research prevailed, culminating in the introduction of biological drugs in the clinical setting. Since the beginning of the 21st century, traditional definition criteria have been challenged by holistic criteria in an effort to seek a still unattained cure. Centuries of worldwide efforts on IBD etiology and therapy search have culminated in this novel strategy.

## 1. Introduction

In the last 70 years, the scientific community has become progressively aware of a trans-organ pattern of inflammation where non-specific mild (indolent) markers of inflammation have replaced the classically known organ-specific stigmata fully manifesting as redness, swelling, and pain [1]. The apparent driving forces of this phenomenon were listed as: (a) switch from an agriculture-based lifestyle towards an industrial and post-industrial mode, (b) privilege of product quantity over quality, resorting to automated work and frequent night-shifts, and (c) changing fiber-based diets for industrial fast-food [2].

As a barrier organ, the digestive tube was among the first structures to be hit by such changes, translating into the epidemic-like entities of ulcerative colitis (UC) and Crohn’s disease (CD), the two main types of inflammatory bowel diseases (IBD) [3]. The attitudes of investigators towards the novel challenges posed by the spreading of IBD (mostly but not exclusively in the Western regions) can be subdivided into three temporal phases: the definition of diagnostic criteria, the search for therapy and, ultimately, an attempt to a holistic vision. Here, we address these three temporal phases of the IBD, and discuss the history of this disease and related evolutionary findings across centuries.

## 2. Methods

Articles published in English regarding the history of IBD diagnosis, the evolution of therapy, and the role of IBD in the context of systemic inflammation were identified through MEDLINE, SCOPUS, ISI-Web of Knowledge, and EMBASE searches using the terms “ulcerative colitis”, “Crohn’s disease”, “inflammatory bowel disease”, “history”, “diagnosis”, “therapy”, and “interactome”. The final search date was 30 May, 2019. Reference lists from published articles were also employed. The titles of these publications and their abstracts were analyzed in order to eliminate duplicates and irrelevant articles. Two authors (G.C.A. and D.G.R.) independently reviewed the literature search results and selected the relevant studies. The full text of all retrieved papers was also assessed to determine whether the inclusion criteria were met.

## 3. Results and Discussion

### 3.1. The History of the Search for Inflammatory Bowel Diseases (IBD) Etiology

The first report of a deadly bowel disease which corresponds to what is now known as UC was done by Matthew Baillie in 1793 [4]. Later, the name “ulcerative colitis” was coined by Samuel Wilks in 1859 in London [5] in a patient with bowel disease which, today, would probably be labeled as CD [6]. In 1907, John Percy Lockhart-Mummery used, for the first time, an electrically illuminated endoscope, designed to reach the sigmoid colon, and reported a malignancy in 7 out of 36 UC patients [7].

Thereafter, transmissible agents [8] were eagerly sought to explain the breakout of IBD, this bias being justified, among other observations, by case-series reporting inter-spouse transmission of CD [9]. No doubt, *Mycobacterium tuberculosis* was the most searched for years. The presence of granulomatous lesions in CD, and the surprising, though erratic, response of cohorts of CD patients to anti-tuberculosis chemotherapeutics justified these conjectures [10]. In 1920, Jacob Arnold Bargen of the Mayo Clinic studied in depth the role of *Diplostreptococci* as causative agents of UC. He repeatedly found *Diplostreptococci* in rectal ulcers of UC patients [11] and could induce colitis in rabbits inoculated with this bacterium [12]. Other microorganisms supposedly implicated as etiologic agents of IBD were *Bacteroides fragilis*, *Bacteroides necrophorum*, *Helicobacter hepaticus* or *pylori*, *Listeria*, *Pseudomonas maltophilia*, pathogenic *Escherichia coli*, *Chlamydia*, *Shigella*, *Coxsakie* A, B, *Wolinella*, Norwalk virus, Polio virus, Reovirus, herpes virus, Paramyxovirus, and Influenze B, as well as the loss of helminths [13].

In 1947, Meyer and Gellhorn proposed that UC was caused by a reduction of the mucus layer above the enterocytes, due to an increase in the lysozyme enzymes, with consequent contact of the luminal bacteria with intestinal mucosa resorting to an inflammatory reaction [14]. Neurosis, food, and pollen allergy [15] were further hypothesized as UC triggers. Moreover, allergy to cow milk was considered as a cause of UC onset [16].

For around twenty years, the “hygiene hypothesis” argued that the increased incidence of allergic and dysimmune diseases could be a consequence of the decreased incidence of infectious diseases (e.g., tuberculosis) in the Western world, mirroring an improved hygiene: the immune system, no longer committed to defense against infectious agents, would be allowed to face commensal microorganisms, resulting in an inappropriate and prolonged inflammatory state [17].

UC has also been considered for many years as a psychosomatic disease [18], with the identification of a specific psychologic profile predisposing to the onset of UC, a tenet heralded ever since the 1930’s. Unfortunately, most of the studies included no control group, and relied on results to draw conclusions while mixed entry criteria generated confusion [18].

The first paper to report an involvement of autoimmunity in the pathogenesis of UC was published by Broberger and Perlmann [19]. In the absence of supporting evidence, this claim did not receive further attention. The fundamental role of genetics as a contributing factor in IBD has become evident when pioneering population studies confirmed the increased incidence in relatives (up to the third degree) of the affected individuals [20]. Through genome-wide association studies (GWAS), more than 230 genes associated with an increased risk of developing IBD were discovered, most of which were shared by both CD and UC [21]. Notably, most of these encode proteins involved in the response to microorganisms.

In 1997, Roediger et al. highlighted the dietary intake of sulfur compounds as a causal factor of UC [22]. In the last few years, the rising incidence of IBD in developing countries has been attributed to the worldwide spread of a Western-style diet (rich in fats, sugars, and refined foods and poor in vegetables, fruits, whole wheat, and nuts), with the intestinal microbiome mediating proinflammatory effects [23].

The human microbiota consists of a wide variety of microorganisms. Every human being harbors between 10 trillion and 100 trillion microbial cells. The indigenous gastrointestinal (GI) tract microflora has relevant effects on the anatomical, physiological, and immunological development of the host. Understanding of human microbiota has evolved over the past years. Culture-independent assays have shown that, although 98% of the GI microbiota is composed of four phyla of bacteria (Firmicutes, Bacteroidetes, Proteobacteria, and Actinobacteria), the majority are from either Firmicutes or Bacteroidetes. Interest in this field has grown and intestinal microbiota and microbiome have become a key topic of investigation in several GI diseases. In the quest for the cause of IBD, the involvement of several bacterial species as well as a decrease in diversity of the intestinal microbiome (dysbiosis), with a reduction of 25% in the bacterial species have been found [24].

#### 3.1.1. The Role of Genetics in the Pathogenesis

Further information on IBD pathogenesis was obtained by genetic studies. More than 230 genetic loci have been identified as associated with an increased risk of developing IBD. The majority of IBD susceptibility genes are linked to pathways involved in immune–microbe interactions and include: microbial detection, immune activation and suppression, and fucosylation of the mucosal epithelium. Some of these genes encode for proteins involved in bacterial recognition, including the interleukin (IL)-23 receptor (IL-23R), the autophagy-related protein 16-1 (ATG16L1) and the IL-10 receptor (IL-10R) [25].

The nucleotide-binding oligomerization domain (NOD)-like receptors (NLRs) are a family of evolutionarily conserved intracellular sensors that recognize a wide range of microbial- and damage-associated signals during infection and inflammation. Upon activation, these innate immune receptors can lead to nuclear factor (NF)-κB and mitogen-activated protein kinase (MAPK) signaling or inflammasome cascades [26]. NOD2 is a cytosolic pattern recognition receptor that senses muramyl dipeptide (MDP), a component of peptidoglycan found in the cell wall of both Gram-positive and Gram-negative bacteria [27]. Mutations are thought to result in “loss of function” and cause defective bacterial sensing. Up to 40% of CD patients have at least one allele mutated in *NOD2*, while mutations in both *NOD2* alleles are found in 10% of these [28]. Upon activation, *NOD2* signaling is mediated by Rip2 kinase, which activates NF-κB and MAPK leading to increased immune gene expression and inflammation. These observations suggest that innate immune responses to bacteria are a key element in the pathogenesis of CD. Furthermore, patients with *NOD2* mutations have reduced defensin production and secretion by Paneth cells, increased T cell and humoral immune responses and, probably, a loss of tolerance to the commensal gut microbiota [29]. *NOD2* is also involved in other cellular defense mechanisms, such as autophagy, in which MDP sensing by *NOD2* induces recruitment of the autophagy protein ATG16L1 to the bacterial entry site in the plasma membrane [30]. Indeed, the CD-associated frameshift mutation of *NOD2* fails to induce ATG16L1 recruitment and results in an incomplete autophagosome formation.

#### 3.1.2. Changes in Epidemiology and Implications for Pathogenesis

The epidemics-like pattern of IBD worldwide in the last 30 years, suggested that a single genetic mutation cannot be the cause of the disease. It is estimated that currently >3 million individuals have IBD in Europe, and 5 million worldwide. A time-trend analysis has shown that 75% of CD studies and 60% of UC studies reported a statistically significant increasing incidence [31]. Rapid industrialization and urbanity of wide areas in the Eastern World coincided with increasing incidence and prevalence of IBD. Recent studies have reported an IBD incidence of 1.37 × 10^5^ in Asia and of 3.4 × 10^5^ in China [32]. These data indicate a rising trend, if compared with the traditional incidence of 0.60–3.44 × 10^5^. As early as 2015, the reasons for this escalation were listed as life westernization, use of appendectomy, milk formula feeding, and changing diets [33]. The incidence of pediatric IBD (CD) in the South Island of New Zealand has long been the matter of scrutiny. Data from a recent study [34] have confirmed an epidemics-like behavior of CD in the area, with one of the highest incidence peaks worldwide, probably caused by reduced bioavailability of vitamin D.

Sources of relevant information regarding IBD are studies of migration. In Canada, Eastern families which adopted a Western lifestyle achieved an IBD incidence rate that closely matched those of Canadian inhabitants; children seemed to be most sensitive to local injuring factors [35]. Cuban exiles rejoining their families in Florida have been described to have a “North American” IBD risk. Interestingly, the authors of the survey highlighted a progressive decrease of the lag time between arrival to Florida and IBD onset [36]. We further hypothesized that a worsening anxious mood of the migrants in response to the rapid changes of the social conditions in the USA, could be responsible for this decrease [37]. Studies of IBD dynamics have so far raised more questions than answers, and it is with genuine anticipation that we reappraised a few recent studies of urbanization of IBD patients, emphasizing a role for microbiome changes. Transitioning from rural to metropolitan life, the switch from fresh prevalently vegetarian food to elaborate meat recipes, and changes of feeding times due to work shift, may have had a deep impact on microbiome, which did not keep pace with the rapid changes [38].

#### 3.1.3. Impact of Diet on Incidence and Course

Compared with healthy controls, CD patients have a lower vegetable and fruit intake, an increased consumption of both processed low fiber bread (white bread), and high-sugar foods. When patients with CD or UC were sub-grouped according to butyrate-acetoacetate Coenzyme A (CoA)-transferase (*BCoAT*) gene content, patients with CD and high *BCoAT* gene concentration had a larger intake of nuts than those with low *BCoAT* levels, whereas no dietary changes were found in patients with UC. When dietary habits were compared, major significant differences between healthy controls and CD patients with low *BCoAT* gene content were observed, with the latter showing reduced intake of certain foods containing fibers such as vegetables, fruits, cereals, brown/whole meal bread, and nuts, and increased intake of high-sugar foods and white bread. Accordingly, inflammation levels, disease-related changes in microbiota composition, and decreased percentage of butyrate-producers were greater in patients with CD having low *BCoAT* gene content than those with high *BCoAT* gene content. This suggests that microbiota modulation, either achieved directly or promoted through the use of prebiotics, might provide more consistent and efficient changes in butyrate concentrations in the gut compared to direct butyrate administration [39].

### 3.2. The History of the Therapy for IBD

At the beginning of the 20th century, the treatment of colitis consisted of bed rest and colon irrigation. For acute cases, an alkaline lotion, such as glycerin thymol 15%, or sodium bicarbonate or, in case of severe ulcerations, hydrogen peroxide [6], was used. One of the first attempts at medical treatment of UC was performed by Robert Fulton Weir in 1902 [40]. He performed an appendicostomy to irrigate the colon with potassium permanganate, hypothesizing an infectious origin of the colitis.

For some years, several vaccines (for example against *Diplostreptococci* [15] or typhus [41]) were evaluated, without success. The first effective medical therapy for UC came with the discovery of sulfonamides in 1938, and with the use of the first antibiotics such as penicillin in 1946, revitalizing the theory of an infectious cause of the disease [41].

Sulphasalazine, devised by Dr. Nana Svartz for the treatment of ‘infective polyarthritis’, was used in the treatment of IBD for more than 70 years. Several controlled trials have shown that sulphasalazine induced remissions in 50% to 75% of patients with active UC. Relapses were five times more likely in untreated patients. Sulphasalazine is less effective in the case of CD, where it exerts only a transient benefit in patients with active colonic disease and fails to prevent relapse or recurrence. Sulphasalazine is absorbed from the small intestine, re-excreted in bile, and carried to the colon, where its azo bond is split by bacteria to release sulphapyridine, which is absorbed and is responsible for most of the drug’s side effects, and 5-aminosalicylic acid (mesalazine), which is the active therapeutic moiety of the drug and exerts a beneficial topical action on the colonic mucosa. Although side effects are common, these are mainly reversible and not serious. Those related to high concentrations of sulphapyridine and to poor acetylation of the drug include GI intolerance, malaise, headache, arthralgia, drug fever, effects on red blood cells, and reversible male infertility. More serious, idiosyncratic side effects are skin rashes, leukopenia, and agranulocytosis. Rarely, neurotoxicity, hepatotoxicity, polyarteritis, pulmonary fibrosis, a lupus-like syndrome, and hemorrhagic colitis, surge. It is possible to desensitize most patients with drug-induced skin rashes. A number of less toxic alternatives to sulphasalazine have been devised. They either convey 5-aminosalicylic acid in a coated tablet to the colon or, when conjugated to a non-toxic carrier, release the drug by bacterial cleavage [42].

The good response of UC to adrenocorticotropic hormone (ACTH) and adrenal corticosteroids in the 1950s [43], in addition to changing the natural history of the disease, opened new horizons on its immunological etiology. The 1940s witnessed men’s struggle for effective pharmaceuticals, somewhat leaving aside the uncertainties over the true disease causes and emphasizing the inflammatory nature of IBD. At that time, intravenous corticosteroids were shown to be clearly better than placebo in the control of UC [44] and various aminosalicylic acid preparations proved effective as maintenance drugs [45]. Brooke et al. [46], pioneering the thiopurines, were unable to document an effect of these drugs on the long-term disease course, yet they recommended the thiopurines [47] in hastening remission. On the other hand, work at the New York Mt Sinai Hospital, did demonstrate a role of azathioprine in achieving and solidly maintaining UC remission. The anecdotic use of 6-mercaptopurine for UC has been reported as early as 1962 [46]. For roughly 30 years, the evidence of the effectiveness of azathioprine/6-mercaptopurine for this disorder has been based on withdrawal experiments [48,49]. Focusing on CD, a pivotal double-blind study performed by Present et al. in 1980, demonstrated the activity of 6-mercaptopurine in fistula closure and steroid sparing for patients with severe disease [50].

Finally, in the 1990s, the sequential use of the transplant drug cyclosporine [51], followed by a long-term thiopurine, ameliorated the course of UC, a noteworthy data if one recalls that, in the 1950s, this disease was expected to be fatal for 30%–40% of those affected [52]. Ultimately, in the 1990s, various formulations of monoclonal antibodies, hopefully capable of blocking those pro-inflammatory cytokines (tumor necrosis factor (TNF), IL-23) that were chorally (non-selectively) addressed by drugs of the cyclosporine generation, were made available to clinicians [53].

Early immunology research, conducted at the Kennedy Institute of Rheumatology in London, demonstrated that TNF, among other cytokines and chemokines, was a primary driver of the inflammatory responses observed in arthritic joints of animal models and in humans. Feldmann et al. “dissected” the synovial tissue and components of the synovial fluid and described elevated expression of TNF in both synovial tissue and synovial fluid of inflamed joints [54]. At Centocor, Knight et al. developed the chimeric anti-TNF monoclonal antibody called infliximab [55]. The research team at the Kennedy Institute of Rheumatology was able to treat, for the first time, rheumatoid arthritis patients with infliximab in a randomized controlled trial [56].

Infliximab was also the first monoclonal antibody available for the treatment of IBD. In fact, it was the first anti-TNF antibody to obtain market authorization for CD in the USA in 1998 and in the EU in 1999. With its 75% human component and a 25% murine component, this chimeric antibody binds the pro-inflammatory protein TNF, both in its circulating and transmembrane form [57]. Infliximab has been shown, since the first trials [58], to be effective in UC even in the most difficult situations of steroid-refractoriness, matching the efficacy of cyclosporine [59]. Unfortunately, the murine component of this hybrid construct has been found to lead, over time, to the production of a significant amount of anti-infliximab antibodies. Thus, combination therapy with thiopurines is recommended in these cases [60]. The standard induction schedule for infliximab, comprising three doses of 5 mg/kg given at weeks 0, 2, and 6, has been derived from studies of CD and rheumatoid arthritis. However, these diseases differ in their biology and inflammatory disease burden in patients with acute severe colitis. A retrospective study by Gibson and colleagues showed that an accelerated infliximab induction schedule within four weeks reduced early colectomy rates at one month compared with standard induction treatment over six weeks [61].

Adalimumab was the first fully humanized monoclonal antibody to be introduced in UC therapy, yet the efficacy was hardly better than placebo in the induction of remission in moderately to severely active forms [62]. Golimumab has been the latest anti-TNF antibody to be introduced for the treatment of UC and is administered subcutaneously every 28 days. It has been shown to be significantly better than placebo in inducing and maintaining remission in UC, without the need for combination therapy with thiopurines [63,64].

The introduction of vedolizumab (which can be considered the “offspring” of natalizumab) marked a breakthrough for the treatment of IBD, as it was the first biological drug selectively acting on the gut. It blocks the α4β7 integrin, thus preventing leukocyte diapedesis from blood vessels to the intestinal mucosa. Its advantages are a prolonged maintenance of remission and a low rate of side effects (intestinal and upper respiratory tract infections). Despite being given intravenously, vedolizumab confers a long-term response latency [65].

Recently, small molecules screening for an IBD cure has received much attention. Unlike biological drugs (which are proteins), small molecules can be taken orally, and have refined bioavailability, making blood levels determination unnecessary. For instance, tofacitinib is currently available on the market. It is a non-selective inhibitor of the intracellular Janus kinase (JAK) 1/3 signaling cascade that has given rapid efficacy results in UC either as a primary indication, or as a secondary resort anti-TNF therapy. The enthusiasm induced by these results is, however, limited by a significant side effect rate (in particular Herpes zoster manifestations, and pulmonary embolism) [66].

Naïve T lymphocytes play a key role in immune surveillance. Activation of these lymphocytes occurs in secondary lymphoid organs, such as spleen, lymph nodes, and Peyer’s patches. The chemoattractant sphingosine 1-phosphate (S1P) guides lymphocyte circulation through these lymphoid organs in a gradient-dependent manner. Ozanimod is an oral S1P agonist that was previously tested in patients with relapsing multiple sclerosis. In a double-blind, phase II randomized controlled trial (RCT), the efficacy and safety of ozanimod in patients with moderate-to-severe UC was evaluated. Clinical remission at week 8 was observed in 16% of patients receiving ozanimod 1 mg once a day (qd), 14% of patients receiving ozanimod 0.5 mg qd, and in 6% of patients receiving placebo. The difference between the 1.0 mg and placebo groups was statistically significant. Furthermore, patients receiving ozanimod 1 mg once qd achieved histologic remission more frequently than those receiving placebo (22% versus 11%). Adverse events were similar in all groups. Phase III trials for CD (ClinicalTrials.gov identifiers: NCT03 440372, NCT03440385, and NCT03464097) and UC (ClinicalTrials.gov identifiers: NCT02435992 and NCT02531126) are ongoing [65]. The heterodimeric pro-inflammatory cytokines IL-12 and IL-23 induce a TH1 and TH17 cell response, respectively. Ustekinumab is a monoclonal antibody directed against the p40 subunit, therefore blocking the biologic activity of IL-12 and IL-23 simultaneously. The UNITI program recruited patients with CD and consisted of two phase III induction RCT (UNITI-1 and UNITI-2) and one phase III maintenance RCT (IM-UNITI). Ustekinumab was efficient both in the induction phase and in the maintenance phase in patients with CD. The safety profile of this drug was acceptable. A phase III induction RCT in patients with moderate-to-severe UC (UNIFI trial) met the primary endpoint of clinical remission at week 8. Patients were also given a single dose of intravenous ustekinumab 130 mg, ustekinumab 6 mg per kilogram of body weight, or placebo. After 8 weeks, 15.6%, 15.5%, and 5.3% of the patients were in clinical remission, respectively. A significant decrease in fecal calprotectin was also observed in patients who received ustekinumab [67].

The increasing appreciation of the multiple causality of IBD has warranted the design of the association of two biological drugs that act on different pathways. On this line, vedolizumab, with its selective action at the level of the intestine, was proposed as the ideal pivot for this strategy: initial reports from patients exposed to a combination of dual biological therapy have described a satisfactory response rate in the presence of an acceptable toxicity [68].

#### 3.2.1. Relationship between Genetics and Response to Therapy

Comparison of genotypes between responders and non-responders revealed a significant difference in the Fas ligand single-nucleotide polymorphism (SNP) rs763110 genotypes. Patients with a CC genotype (compared to those with a TC or TT genotype) were more likely to be non-responders to anti-TNF treatment (*p* = 0.016; odds ratio (OR) = 0.31). Abnormal regulation of apoptosis is one of the mechanisms of CD pathogenesis. The extrinsic pathway is controlled through plasma membrane receptors belonging to the TNF receptor superfamily that includes, among others, the Fas/Fas ligand which has been implicated in IBD pathogenesis. Higher basal expression of the Fas ligand has been significantly associated with the C allele compared with the T allele of this SNP [69]. Jürgens et al. demonstrated that patients homozygous for IL-23 receptor SNPs, which increased the risk of IBD onset, were more likely to respond to anti-TNF therapy compared to patients with IL-23 receptor SNPs that had decreased risk [70].

A polymorphism of CCL2, a chemokine part of the IL-23 pathway that decreases T-cell migration and impacts downstream on TNF-α levels, decreased the risk of durable response. It is possible that polymorphisms in SNP decrease the effect of this protective chemokine. Suppressor of cytokine Signaling 1 (SOCS1), a component of the IL-12 pathway, was associated with a higher likelihood of durable response. PTPN22 (rs6679677) was associated with 2.26-fold increased odds of primary non-response to anti-TNF therapy [71].

#### 3.2.2. Attempt to Target Microbiota

The term “microbiome” [72] is commonly used to indicate any microbe population that indwells the so-called barrier organs. Because of the huge number of bacteria, fungi, and bacteriophages in the bowel, the multiple functions of the gut, and the involvement of its disorders into a huge series of human diseases, the gut-associated microbiome has received renewed attention lately. We now know that the gut microbiome components encompass 10 times the number of human cells in the body, and the relevant genetic material is 100 times greater [73].

At gut level, a correct immune balance can be maintained only on the background of a “normal” composition of the microbiome. Abnormal communication between gut microbial communities and the mucosal immune system has been identified as the core defect that leads to chronic inflammation [74]. An alteration in the diversity and composition of the gut microbiome (dysbiosis), rather than the presence of specific pathogens, likely plays a critical role in IBD pathogenesis. Several studies have shown that there is a significant reduction in the diversity of stool microbiome of individuals with IBD: 25% fewer genes were detected in the fecal samples of IBD patients than in those of control patients. The majority of IBD susceptibility genes (for example *NOD2*, *ATG16L1*, and *IL-23R* genes) are linked to pathways involved in immune–microbe interactions [75].

Mesalazine inhibits the growth of *Mycobacterium avium* subspecies *paratuberculosis*, which has been reported to be intimately linked to the etiology of CD in a dose-dependent manner in vitro. Furthermore, it has been reported that mesalazine downregulates the expression of genes associated with bacterial invasiveness and antibiotic resistance of *Salmonella enterica* serovar *Typhimurium*, which could promote the onset of IBD after its infection. Furthermore, mesalazine inhibits the growth of sulfate-reducing bacteria and suppresses sulfide production in IBD.

Anti-TNF-α antibody therapy has been reported to affect the gut microbiota. For instance, the abundance of *Faecalibacterium prausnitzii* increased in responders during the induction of anti-TNF-α antibody therapy. Another study reported that the relative number of *Escherichia coli* in CD significantly decreased three months after the start of anti-TNF-α antibody therapy [76].

Interestingly, thiopurines were found to inhibit the growth of Mycobacterium avium subspecies paratuberculosis in vitro [77]. Another study reported that thiopurine use significantly reduced the bacterial diversity and richness in fecal samples in IBD, when compared to other drugs, including anti-TNF-α antibodies, mesalazine, and corticosteroids [78].

In a prospectively recruited cohort of patients with IBD initiating vedolizumab therapy, the relationship between gut microbial taxonomic composition and function and response to vedolizumab therapy was investigated. Eighty-five patients (42 with CD and 43 with UC) were included. Community α diversity at baseline was significantly higher in CD patients who achieved clinical remission at week 14 compared to those who did not achieve this endpoint. Among responders, two taxa species, *Roseburia inulinivorans* and Burkholderiales, were significantly more abundant at baseline than at week 14. No significant association was found between gut microbiota and clinical remission at week 14 in UC patients [79].

It has been reported that reduced Firmicutes abundance was correlated with a shorter time to relapse after infliximab withdrawal. The abundance of six clades of bacteria, including *Eubacterium rectale* and *Bifidobacterium* spp., predicted the response to anti-TNF medication in pediatric IBD patients [80]. We investigated, in a prospective study, the gut microbiota profile before and after adalimumab therapy, in patients with CD. Considering bacterial phyla, Proteobacteria decreased significantly in patients in whom therapeutic success was obtained, passing from a value of 15.8% to 6.8%, while in non-responder patients, their percentage did not change. Considering the Lachnospiraceae family, in patients with normalization of C reactive protein after six months of adalimumab therapy, it increased from 16.6% to 23.9% (*p* = 0.049). We concluded that in patients who respond to adalimumab therapy, by decreasing inflammation, there is a trend to restore the intestinal eubiosis [81]. Thus, gut microbiota may provide potential biomarkers for monitoring and predicting IBD treatment outcomes.

Since the discovery of the pivotal role of microbiota in inflammatory diseases, an increasing number of studies has been performed regarding the efficacy of fecal microbiota transplantation (FMT) in intestinal and extra-intestinal diseases [82]. In the RCT conducted by Moayyedi et al. [83], the authors found that early diagnosis of UC might result in better outcomes after FMT. This suggested a potential window of opportunity for FMT after diagnosis of UC. Accordingly, Paramsothy et al. [84] reported that less severe grades of endoscopic inflammation may be a potential predictor of FMT response. Moreover, the increase in *Clostridium* clusters IV and XVIII and the high microbial diversity were identified as predictors of FMT response. Conversely, the presence of *Fusobacterium spp*. and *Sutterella* spp. were associated with non-response to FMT.

### 3.3. An Attempt to a Holistic Vision

Grown in number and activity, the anti-cytokine monoclonals are still the preferred treatment regimen of most clinicians against IBD. However, these drugs may fail to terminate the disease and may activate paradoxical immune diseases [85]. This suggests that IBD is part of an autoimmune disease cluster, sharing intertwined regulatory rules with the other members of the cluster (rheumatism, psoriasis, multiple sclerosis, and others): the dual action of a monoclonal drug may silence one pro-inflammatory pathway, while disturbing other protective circuits.

Two pieces of evidence must be added to close the circle of the above statements. Firstly, the pathophysiology of IBD is extraordinarily active beyond the bowel walls, triggering an array of inflammatory manifestations that can involve joints, biliary tree, skin, and eyes (the well-known extraintestinal manifestations of IBD) [86]. Secondly, refinement of molecular biology techniques has led to the revolutionary demonstration of the involvement of gut microbiome, a universe of 10^16^ bacterial/fungal/viral species, in IBD. This huge metagenome can produce substrates that elude our limited immunologic repertoire and can ignite inflammation coping with abrupt diet changes but may also activate a pro-inflammatory phylum responsible for intestinal or extraintestinal pathology [87,88].

At present, therapies that target molecular pathways do not provide uniform benefits for all patients. New transformative therapies for autoimmune and inflammatory diseases require greater molecular understanding of patient subsets and the ability to personalize targeted agents [89]. The integrated “omics” approach, rendered possible through the analysis of a collection of datasets (termed “big data”), shows promise in designing IBD patient-tailored diagnosis and therapeutic opportunities. The factors previously mentioned as associated with IBD, i.e., the environment, the genes, the microbiota, and the immune systems, may all be critically important but, given their individual complexity, a functionally integrated vision may be required. The intricate and reciprocal interaction among these entities, through the so-called ‘IBD interactome’, results in the emergence of IBD, or more appropriately the ‘IBD integrome’ [90,91]. In its broadest definition, an interactome indicates the whole set of molecular interactions in a given cell. Such interactions may regard antigens (for example proteins) but can also describe sets of indirect interactions among genes [92]. Regarding IBD, an interactome may also be intended to identify sets of mechanisms that are indispensable for a disease to be maintained, and, ideally, families of drug molecules capable of targeting those mechanisms leading to disease termination. As far as the latter is concerned, there is wide availability of drugs that may not work when applied in a general context, but may target specific pathways, and are thus awaiting proper patient classifying algorithms to be tested.

Genome-wide associated studies of omics data have revealed modules of disease-associated genes that have been used to obtain a molecular understanding of disease mechanisms [93]. In addition to gene level, “exposome” or “environome” analysis complements the genome by providing a comprehensive description of lifelong exposure history: it could be useful for better delineating the causes and prevention of human disease [94]. It is expected that although the genome is the blueprint of an individual, its analysis with that of the other “omes” such as the DNA methylome, transcriptome, proteome, and metabolome, will further provide a dynamic assessment of the physiology and health state of an individual (the disease interactome) [95]. Existing drugs, targeting specific biological pathways identified in the interactome analysis, could be tried first in IBD models, then in clinical practice thereafter [96]. To better define interactome in IBD, a new strategy should focus on pathways of inflammation, and integrate data from clinical studies obtained by applying biologic treatments. There is also an urgent need for bioinformatic tools to analyze and integrate the huge quantity of data derived from “omics” approach in order to obtain comprehensive molecular pathways for IBD. This approach could lead to identification of IBD molecular subtypes and correlation with clinical phenotypes, and aid in the discovery of compounds to finally have better disease control [97]. The future IBD therapeutics should be developed on the basis of targeting, through a systems biology approach, the central hubs in the IBD network [98].

## 4. Conclusions

According to today’s vision, the IBD inflammation is not “simply” a universal, highly conserved force. In any given patient, inflammation may bear the patient’s personal signature, with the microbiome mainly imprinting its personal touch. If looked at closely, the microbiome is a “transponder” conveying, to the inner gut, information from the surrounding environment. Easy to predict, the physiologic “transponder” [91] will be mostly fed by information on diet, establishing a continuous information axis between the body tending to stability and the variations outside, that call for inflammation remodeling. Lingering gut inflammation may thus be conceived as the factor keeping our machinery connected to “what’s going on out there”. Though important, diet composition may be but a part of the “world out there”: working times, environment, including an interconnected network of variables such as feeding rhythm, noise pollution, breaking-in artificial light, and scents/odors, all contribute to the composite message influencing our sensors daily. The anxiety-depression-inflammation equation may be a reasonable interpretation key of the above premises [85].

A holistic approach could bear more chance of success than single switch inactivation one at a time, as pursued until now, for example, with biological drugs.
